# A high‐throughput method to quantify feeding rates in aquatic organisms: A case study with *Daphnia*

**DOI:** 10.1002/ece3.6352

**Published:** 2020-06-16

**Authors:** Jessica L. Hite, Alaina C. Pfenning‐Butterworth, Rachel E. Vetter, Clayton E. Cressler

**Affiliations:** ^1^ School of Biological Sciences University of Nebraska Lincoln Nebraska USA

**Keywords:** aquatic herbivore, consumer–resource, *Daphnia*, environmental contaminants, exposure rates, feeding rates, High‐throughput, ingestion rates, predator–prey, transmission

## Abstract

1. Food ingestion is one of the most basic features of all organisms. However, obtaining precise—and high‐throughput—estimates of feeding rates remains challenging, particularly for small, aquatic herbivores such as zooplankton, snails, and tadpoles. These animals typically consume low volumes of food that are time‐consuming to accurately measure.
2. We extend a standard high‐throughput fluorometry technique, which uses a microplate reader and 96‐well plates, as a practical tool for studies in ecology, evolution, and disease biology. We outline technical and methodological details to optimize quantification of individual feeding rates, improve accuracy, and minimize sampling error.
3. This high‐throughput assay offers several advantages over previous methods, including i) substantially reduced time allotments per sample to facilitate larger, more efficient experiments; ii) technical replicates; and iii) conversion of in vivo measurements to units (mL^‐1^ hr^‐1^ ind^‐1^) which enables broad‐scale comparisons across an array of taxa and studies.
4. To evaluate the accuracy and feasibility of our approach, we use the zooplankton, *Daphnia dentifera,* as a case study. Our results indicate that this procedure accurately quantifies feeding rates and highlights differences among seven genotypes.
5. The method detailed here has broad applicability to a diverse array of aquatic taxa, their resources, environmental contaminants (e.g., plastics), and infectious agents. We discuss simple extensions to quantify epidemiologically relevant traits, such as pathogen exposure and transmission rates, for infectious agents with oral or trophic transmission.

## Graphical abstract

Ever tried to accurately quantify feeding rates in aquatic herbivores? It's tedious and slow! We extend and refine a high‐throughput method that is commonplace in other fields but relatively underutilized in ecology and evolutionary biology.

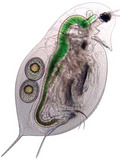

## 1 INTRODUCTION

Energy ingestion (feeding) is arguably the most central biological process. Consumer–resource interactions are the building blocks of ecological food webs and form the cornerstone of ecological theory (Murdoch, Briggs, & Nisbet, 2003; de Roos & Persson, 2013). Feeding behavior is highly plastic, changing across ontogeny (Rudolf & Rasmussen, 2013; ten Brink & de Roos, 2017) and in response to threats from predators (Costa & Vonesh, 2013; Matassa, Donelan, Luttbeg, & Trussell, 2016), infectious agents (Bernardo, & Singer, 2017Hite, Pfenning, & Cressler, 2020), resources (Mandal, Abbott Wilkins, & Shurin, 2018, Penczykowski et al., 2014; Sarnelle & Wilson, 2008), and environmental contaminants such as heavy metals and microplastics (Carrasco et al., 2019; Cole et al., 2013; Setala, Norkko, & Lehtiniemi, 2016). Changes in feeding rates can, therefore, reveal mechanistic connections between behavior, physiology, and immunology with applications for multiple disciplines.

Measuring feeding rates, however, is typically a time‐consuming and logistically challenging endeavor. This is especially true for small aquatic herbivores such as zooplankton, snails, and tadpoles. These animals typically consume low volumes of food that are time‐consuming to accurately measure. Previous efforts have largely relied on time‐intensive processes such as electronic particle counting systems (Seale, 1982), single‐channel fluorometers (Penczykowski et al., 2014), dry mass and cell counts (Sarnelle & Wilson, 2008), radioactive labels (Hong, Burford, Ralph, Udy, & Doblin, 2013), and dyes or beads (Cole et al., 2013; Setala et al., 2016). While these methods are widely used, each carries unique disadvantages, and all suffer from pronounced time constraints that hinder high‐throughput experiments. For instance, processing time for a *single*‐channel fluorometer is approximately two min. per sample (J. L. Hite, unpublished data).

To overcome these limitations—and highlight a practical tool for studies in aquatic ecology, disease ecology, and evolutionary epidemiology—we present a standard high‐throughput fluorometry method that uses a microplate reader and 96‐well plates (Garbutt & Little, 2014; Nasser & Lynch, 2016; Ogonowski, Schür, Jarsén, & Gorokhova, 2016). We provide specific technical and methodological protocols to optimize quantification of individual feeding rates, improve accuracy, and minimize sampling error. The approach detailed here offers three key advantages over previous methods. First, it is precise and high‐throughput and substantially reduces time allotments per sample. Processing time runs approximately 15 min, from pipetting (with a multi‐channel pipette), to analyzing and exporting the data for a 96‐well plate of samples. In other words, instead of a single measurement taking two min. per sample, one can read a 96‐well plate in two min.. Second, unlike previous methods outlined above, this assay includes technical replicates. Third, conversion of in vivo measurements to units (mL^‐1^ hr^‐1^ ind^‐1^), which facilitates quantitative comparisons across a broad array of taxa and studies.

## 2 MATERIALS AND METHODS

This method uses a microplate reader (Tecan©, Maennedorf, Switzlerand) to quantify feeding rates using in vivo narrow‐band fluorometry, a standard and widely used method for accurately measuring chlorophyll‐*a* (Kalaji et al., 2014; Lorenzen, 1966). In brief, the goal is to compare the fluorescence of algae in tubes with animals (consumers) versus the fluorescence of algae in the animal‐free (consumer‐free) controls, following Sarnelle and Wilson (2008). We provide a detailed protocol (and overview of materials, Fig. 2) aimed at improving repeatability and analytical accuracy, while minimizing variation among samples. The most important, but easily overlooked, details include (a) preparing all media in batch cultures and mixing it continuously prior to and throughout distribution to each biological replicate; (b) conducting the assay under minimal light conditions to prevent spurious spikes in fluorescence; (c) ensuring that the ratio of chlorophyll to carbon remains constant across assays; and (d) pair‐matching plate‐specific controls with their respective treatment samples to reduce among‐plate variation.

### 2.1 Cleaning procedures

All glassware and plasticware used in the assays were acid‐cleaned (8%–10% H_2_SO_4_ or HCL) and thoroughly rinsed with deionized water (four times) prior to use. This added precaution helps improve analytical accuracy by removing potential contamination from trace amounts of minerals, metals (e.g., copper), nutrients (e.g., phosphates), or microbes.

### 2.2 Feeding rate assay

**FIGURE 1:**
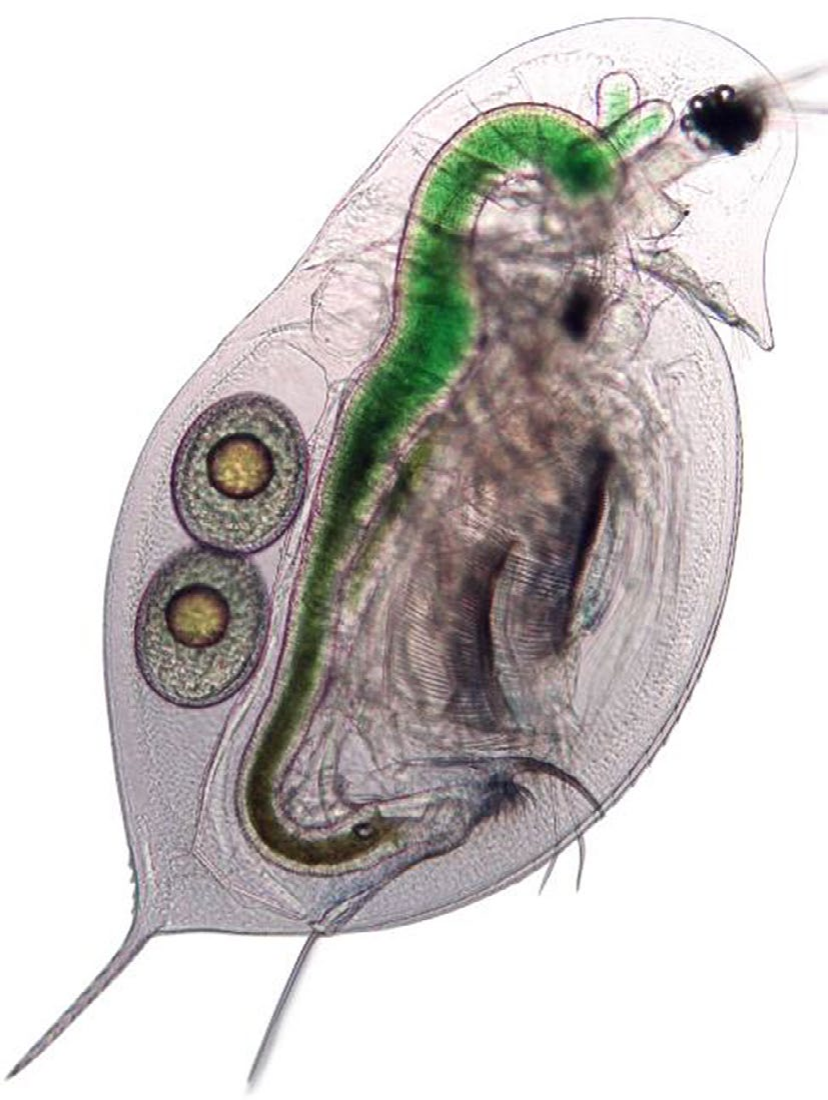
The focal organism, *Daphnia dentifera*. *Daphnia* are small (~1–3 mm) freshwater crustaceans and play a prominent role in many fields including aquatic ecology, life‐history theory, physiology, toxicology, cellular immunology, and disease ecology. Photograph credit: Tara Stewart Merrill

To refine this standard method, we measured feeding rates of 6‐day‐old female *Daphnia dentifera* (Figure 1) from seven different isoclonal lines (hereafter, genotypes) with 30 replicates per genotype and spore level (*n* = 630). Genotypes were originally collected from different lakes in Southern Indiana and Michigan (USA). We previously identified these lines as unique genotypes by comparing alleles at microsatellite loci (Strauss et al., 2017), and other studies have used subsets of these genotypes to examine genotypic variation in epidemiologically and evolutionarily relevant traits, including feeding rates (Shocket et al., 2018; Strauss, Bowling, Duffy, Cáceres, & Hall, 2018; Strauss et al., 2019). These genotypes, therefore, provide an ideal case study to test the accuracy of the method detailed here. Due to logistical (i.e., time) constraints, we conducted the assays over two temporal blocks, block one with five genotypes and block two with four genotypes with two genotypes repeated among blocks to highlight any potential block effects (e.g., from potential variation in algal or spore infectivity). We omitted any negative feeding rates (since these represent technical errors), individuals that died during the assay, and animals later identified as male; male and female *Daphnia* have different feeding rates (Hite et al., 2017).

For the food source, we used batch‐cultured algae (*Ankistrodesmus falcatus*) of the same age and growth stage (the predetermined stationary phase under our specific conditions: 14 days old). We cultured algae in 1 L Erlenmeyer flasks containing 500 ml of high nitrogen COMBO (Kilham, Kreeger, Lynn, Goulden, & Herrera, 1998) under a 16:8 light–dark cycle at 40 ± 5 μmol photon·m−2·s−1 of photosynthetically active radiation (PAR). We inoculated batch cultures with 10 ml of 14‐day‐old *A. falcatus* grown under identical conditions. We made the high nitrogen COMBO with filtered (0.2 μm) and UV‐sterilized DI water (PureLab Ultra, Evoqua Water Technologies) and by doubling the nitrogen in the base media. Since the relationship between chlorophyll fluorescence and carbon content changes throughout algal growth stages, it is crucial to inoculate each flask with algae of the same density, age, and growth stage to ensure identical starting points; these steps ensure consistency in the chlorophyll: carbon ratio (used in calculations below), as well as nutrient (e.g., nitrogen and phosphorus) and lipid content per cell, all of which can affect herbivore feeding rates Halsey & Jones, 2015; Mandal et al., 2018; Sterner, 1993).

We conducted the entire assay (setup to takedown) with the lights off and windows shaded to prevent algal growth or any spurious spikes in algal fluorescence. During the timed feeding rate assay, we moved the centrifuge tubes to a completely dark incubator maintained at 22 ± 1°C. For best practices, we suggest preacclimating animals to the food and temperature conditions used in each assay for at least two days.

**FIGURE 2:**
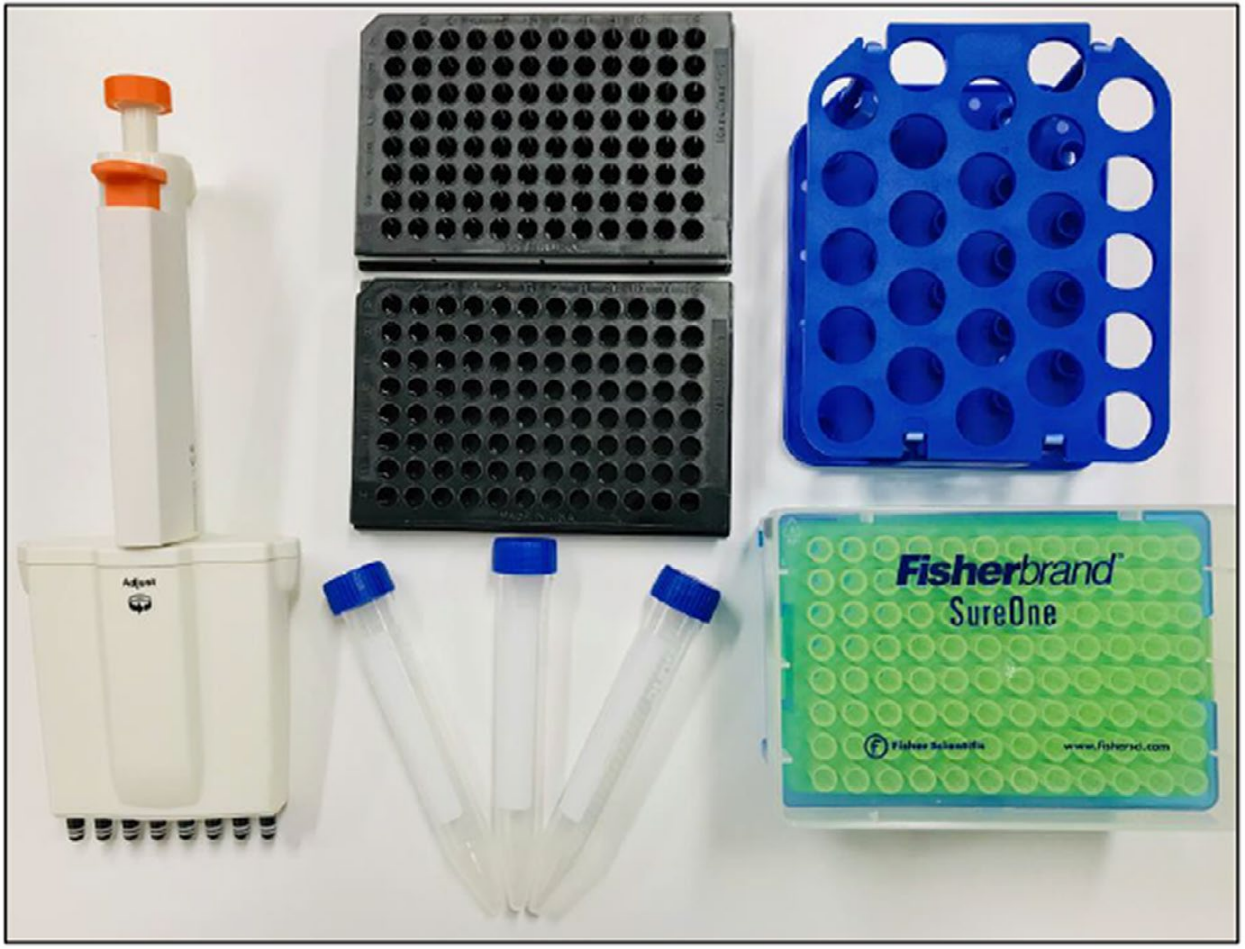
Hardware needed for the high‐throughput method detailed here: multi‐channel pipette with autoclaved pipette tips, black 96‐well plates, 15 ml centrifuge tubes with caps and holder.

Prior to starting the assay, we calculated the entire amount of media needed for the assay and made a large primary solution consisting of COMBO and algal food (1.0 mg dw L^−1^). We determined the relationships between algal density (mg C L^−1^) and optical absorbance (800 nm 1 cm cuvette) using a regression‐based relationship between absorbance and dry mass (a close correlate of carbon). To ensure that the algal food and media remained evenly mixed and consistently distributed for each biological replicate (individual centrifuge tube), we placed the beaker of primary solution on a stir plate (rotating at low‐medium speed). We pipetted 10 ml of media into individual 15 ml centrifuge tubes.

Once all media were distributed, we used glass pipettes to transfer individual *Daphnia* into each centrifuge tube (except, of course, the animal‐free controls), capped the tubes, started the timer, and moved the animals to the incubator. To keep algal food in suspension, we gently inverted tubes every 30 min over the course of the assay. At the end of the assays (seven hrs), we transferred hosts to fresh media and collected a subsample of media from each tube to quantify the remaining food.

### 2.3 In vivo fluorometry

We measured algal fluorescence using narrow‐band fluorometry. We used the multichannel pippette to distribute 200 μL of sample (each with two technical replicates) into wells of a black 96‐well plate (14‐245‐197A Thermo Fisher Scientific No. 7605). We used top read mode with standard initial parameters following Gregor and Maršálek (2005) and Kalaji et al. (2014): excitation wavelength: 485 (20 nm bandwidth); emission wavelength: 665 (10 nm bandwidth); gain (optimal range): 112–120, number of flashes: 30; lag time: 0; integration time: 40 μs; shake duration: 10 s; and temperature: 22.5°C ± 0.5. The particular brand and model of plate reader is nominal. However, the proper filters and narrow bandwidth are crucial.

To minimize noise introduced by among‐read variation (Petersen & Nguyen, 2005; Resch‐Genger et al., 2005), it is crucial that each plate is treated as a block and contains control samples (i.e., media from the consumer‐free replicates) and that the average of these plate‐specific controls is used to calculate feeding rates. That is, in the equations below, the control values come from each plate and are not averaged across all controls. This design reflects a matched‐pairs layout and uses replication within blocks to help tease apart main effects, block effects, and their interaction (Gotelli & Ellison, 2004). This added step is an obvious limitation of this method. However, this is currently the best solution for maximizing the signal‐to‐noise ratio given the extreme sensitivity of modern fluorometers. In Appendix S1, we include R code to facilitate these additional quality control steps.

We calculated the feeding rate of individual *Daphnia*, *f* (L ind^−1^ day^−1^) following Sarnelle and Wilson (2008), by solving for the change in fluorescence, *F*:

(1a) $$\frac{dF(t)}{\mathrm{dt}}=-\frac{f}{V}F\left(t\right)$$

Fluorescence is determined by the biomass of algae in the sample, measured in mg dw L^−1^. Feeding measures the amount of space or volume cleared per unit time. Thus, if *W* is the biomass of algae per L, and *f* is the feeding rate (L time^−1^), then *fW* is the rate at which biomass is removed from the media. Mathematically, this is given by the solution of the ODE:

(1b) $$\frac{dW(t)}{\mathrm{dt}}=-\frac{f}{V}W\left(t\right)$$

Note, you have to divide by volume to get the units right, since volume occurs in the units of both *f* and *W*. We can convert the change in biomass density to change in fluorescence assuming a strong linear relationship between fluorescence and biomass (which we double‐checked using the standard curve of fluorescence against biomass detailed above) then,

F=lW,

where *l* is the slope of the regression. The dynamics of *F* are as follows:

(1c) $$\begin{array}{c} \frac{dF(t)}{\mathrm{dt}}=\frac{d(lW(t))}{\mathrm{dt}}=l\frac{\mathrm{dW}}{\mathrm{dt}}=l\left(-\frac{f}{V}W\left(t\right)\right)\\ =-\frac{f}{V}\left(lW\left(t\right)\right)=-\frac{f}{V}F\left(t\right). \end{array}$$

The solution of this linear differential equation is

(1d) $$F\left(t\right)=F\left(0\right)e^{-\frac{f}{V}t}$$

where *F*(*t*) is the food remaining (i.e., the mean algal fluorescence of the sample at time *t*), *F*(0) is the initial amount of food (i.e., the mean algal fluorescence of the corresponding plate‐specific consumer‐free controls at time = *t*0), *V* is the volume of media (10 ml), and *t* is the length of the assay. Solving for *f*, then:

(1e) $$f=\ln\left(\frac{F(0)}{F(t)}\right)\frac{V}{t}$$

### 2.4 Statistical analysis

To test for differences in feeding rates across genotypes and across temporal blocks, we used analysis of variance (ANOVA) with Type III sum of squares to account for the unbalanced sample sizes. We confirmed that residuals of the feeding rate model did not deviate from normality using visual inspections and the Shapiro–Wilk test (*p* = .14). To highlight differences/similarities among genotypes, we used Tukey's post hoc analyses (multcomp package in R version 3.6.1).

## 3 RESULTS

**FIGURE 3:**
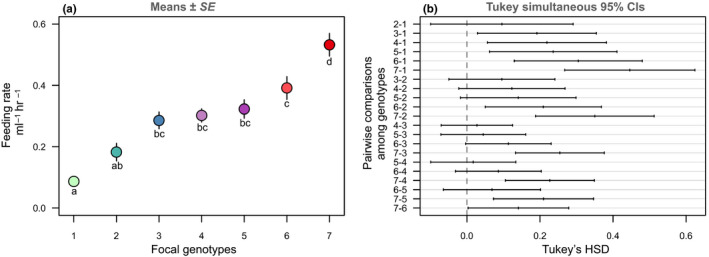
Feeding rates across seven focal genotypes. (a) Points are means (±*SE*) from the feeding rate assays (conducted across two temporal blocks). Lowercase letters represent significant differences among genotypes (based on *post hoc* analyses). (b) *Post hoc* pairwise comparisons with Tukey's adjusted *p*‐values and simultaneous 95% confidence intervals. If an interval does not contain zero, the corresponding means are significantly different

Our method successfully detected differences in feeding rates among the focal genotypes (main effect: $\chi_{6,1}^{2}$ = 65.62 *p* < .0001) and across temporal blocks (*p* < .0001). However, there was no interaction between genotype and temporal block (*p* = .774) and visually illustrating the differences across genotypes and blocks is rather logistically challenging and obscures the main goal here to demonstrate that this method accurately captures similarities/differences in feeding rates. Therefore, we present these main effects averaged across temporal blocks ( $\chi_{6}^{2}$ =  80.99, *p* < .0001, Fig. 3), which is recommended for experiments with simple nested designs (Gotelli & Ellison,^2004^, pgs. 178–182). Both the mean and variation in the feeding rates estimated by this method are congruent with previous estimates of *Daphnia* feeding rates (Garbutt & Little, 2014; Nasser & Lynch, 2016; Sarnelle & Wilson, 2008; Strauss et al., 2019).

## 4 DISCUSSION

This high‐throughput method enables larger, more efficient experiments and can be applied to a wide array of aquatic taxa and experimental designs. Due to its ability to quantify fine‐scale differences in a relatively short amount of time, the high‐throughput fluorometry technique is common in, for example, water quality management (Carstea, Bridgeman, Baker, & Reynolds, 2016; Gregor & Maršálek, 2005), agriculture (Kalaji et al., 2014, 2018), physiology (Bergman Filho, Soares, & Loureiro, 2011; Matoo, Julick, & Montooth, 2019), and medical microbiology (Chinen et al., 2015). To date, these methods and practical tools remain relatively underutilized in aquatic ecology, disease ecology, and evolutionary epidemiology. In extending and refining a standard high‐throughput fluorometry technique, we outlined technical and methodological protocols to optimize quantification of individual feeding rates, improve accuracy, and minimize sampling error.

The feeding rates estimated by this method are congruent with previous estimates of *Daphnia* feeding rates (Garbutt & Little, 2014; Nasser & Lynch, 2016; Sarnelle & Wilson, 2008; Strauss et al., 2019). This method can be extended to account for differences in algal diversity (Gregor & Maršálek, 2005) and extremely low algal concentrations or high turbidity (Chang, Hobson, Burch, & Lin, 2012), as well as environmental stressors including environmental contaminants like microplastics (Carrasco et al., 2019; Cole et al., 2013; Setala et al., 2016).

Additionally, this method also carries applications for disease ecology and evolutionary epidemiology. For instance, using slow‐throughput methods (i.e., single‐channel fluorometers), we previously found that feeding rates of the freshwater zooplankton *Daphnia dentifera* correlate strongly with the consumption of a fungal pathogen, *Metschnikowia bicuspidata* (Hite et al., 2017; Shocket et al., 2018; Strauss et al., 2019), and more detailed counts of stained and filtered fungal spores confirm a strong positive relationship between algae intake and pathogen intake (Strauss et al., 2019).

Such extensions involve adding other batch cultures that contain algae, sample media (e.g., COMBO), and the stressor of interest. Again, to ensure that the media remain evenly mixed and distributed among individual sampling units, it is crucial to keep the primary solution on the stir plate prior to and throughout the distribution step. This step is particularly important when combining this assay with exposure to other environmental contaminants, such as pathogens, which could potentially sink out of solution (Hall, Smyth, et al., 2010). The only additional step involves controls that are animal‐free but include the stressor of interest (e.g., pathogen propagules, microplastics). These samples now replace the pure algae controls in the feeding rate assay and will account for any background fluorescence of the contaminant or infectious agent, which will likely be minimal but nonetheless important, given the sensitivity and high resolution of modern fluorometers.

## CONFLICT OF INTEREST

The authors declare no conflict of interest.

## AUTHOR CONTRIBUTIONS

**Jessica L. Hite:** Conceptualization (lead); data curation (lead); formal analysis (lead); funding acquisition (lead); investigation (lead); methodology (lead); project administration (lead); resources (lead); supervision (lead); validation (lead); visualization (lead); writing–original draft (lead); writing–review and editing (lead). **Alaina C. Pfenning‐Butterworth:** Conceptualization (supporting); data curation (supporting); investigation (supporting); methodology (supporting); project administration (supporting); validation (supporting); writing–review and editing (supporting). **Rachel E. Vetter:** Investigation (supporting); methodology (supporting); project administration (supporting); writing–review and editing (supporting). **Clayton E. Cressler:** Data curation (supporting); formal analysis (supporting); funding acquisition (supporting); methodology (supporting); project administration (supporting); resources (supporting); supervision (supporting); validation (supporting); writing–review and editing (supporting).

### Multiple Badges

This article has been awarded Open Data, Preregistered Badges. All materials and data are publicly accessible via the Open Science Framework at https://doi.org/10.5061/dryad.sqv9s4n1c; https://doi.org/10.5061/dryad.sqv9s4n1c.

## Supporting information

Supplementary Material

## Data Availability

R code used in this manuscript is available in Appendix S1. Data are available at: https://doi.org/10.5061/dryad.sqv9s4n1c.
